# Supplementary material for: A fully automatic AI system for tooth and alveolar bone segmentation from cone-beam CT images
Source: Nat Commun. 2022 Apr 19;13:2096. doi: 10.1038/s41467-022-29637-2 (PMC9018763; doi:10.1038/s41467-022-29637-2)
Supplement: Supplementary file 1 — Supplementary Information [file 41467_2022_29637_MOESM1_ESM.pdf]

Supplementary Information for: A fully automatic AI system for tooth and alveolar bone segmentation from cone-beam CT images

Zhiming Cui[1,2,9,*], Yu Fang[1,*], Lanzhuju Mei[1,*], Bojun Zhang[3,*], Bo Yu[4], Jiameng Liu[1], Caiwen Jiang[1], Yuhang Sun[1], Lei Ma[1], Jiawei Huang[1], Yang Liu[6], Yue Zhao[5,†], Chunfeng Lian[7,†], Zhongxiang Ding[8,†], Min Zhu[3,†], and Dinggang Shen[1,9,†]

[1]School of Biomedical Engineering, ShanghaiTech University, Shanghai, 201210, China
[2]Department of Computer Science, The University of Hong Kong, Hong Kong, 999077, China
[3]Shanghai Ninth People's Hospital, Shanghai Jiao Tong University, Shanghai, 200011, China
[4]School of Public Health, Hangzhou Medical College, Hangzhou, 310013, China
[5]School of Communication and Information Engineering, Chongqing University of Posts and Telecommunications, Chongqing, 400065, China
[6]Department of Orthodontics, Stomatological Hospital of Chongqing Medical University, Chongqing, 401147, China
[7]School of Mathematics and Statistics, Xi'an Jiaotong University, Xi'an, 710049, China
[8]Department of Radiology, Hangzhou First People's Hospital, Zhejiang University, Hangzhou, 310006, China
[9]Shanghai United Imaging Intelligence Co., Ltd., Shanghai, 200030, China
[†]Corresponding authors: Dinggang Shen (dgshen@shanghaitech.edu.cn), Min Zhu (ZHUM1612@sh9hospital.org.cn), Chunfeng Lian (chunfeng.lian@xjtu.edu.cn), Yue Zhao (zhaoyue@cqupt.edu.cn), Zhongxiang Ding (hangzhoudzx73@126.com)
[*]These authors contributed equally to this work

Hierarchical data annotation method

We applied a hierarchical data annotation method to iteratively annotate all the CBCT scans in our dataset. Specifically, we first randomly selected 100 CBCT scans from the dataset, and 3 senior raters manually annotated the label including individual teeth and alveolar bones. This stage of fully manual annotation took about 2 months. Then, we used the 100 annotated CBCT scans to train our proposed system, and tested on the next 600 CBCT scans to produce initial segmentation results. Ten junior raters independently corrected the 600 labeled CBCT images to obtain preliminary results. Next, three senior raters checked the preliminary labeling results, and mode the final decision. The second stage took about 5 days for correction. At last, the 700 annotated CBCT scans were used to train the AI system, which in return tested on the rest 4238 CBCT scans. Again, the final labeling results are corrected and checked by the ten junior raters and three senior raters, which took about 40 days to complete the process. The flow of the hierarchical data annotation method is shown in Supplementary Fig. 1.

Instead, to avoid inter-practitioner's variations in the data annotation process, all CBCT scans were independently labeled or checked by the 3 senior raters. When disagreements occurred among the 3 senior raters, they would have a discussion to make the final decision.

Supplementary Table 1. The data distribution of the abnormalities in the training and testing datasets.

Dental abnormalities	Internal set		External set
	Training dataset (3172 CBCT scans)	Internal testing dataset (1359 CBCT scans)	External testing dataset (407 CBCT scans)
Missing teeth	946	345	137
Misalignment	2351	1085	314
Metal artifacts	435	199	96

Supplementary Table 2. Ablation study of the tooth segmentation on internal and external testing sets.

Class	Model	Internal testing set			External testing set		
		Dice (%)	Sen (%)	ASD (mm)	Dice (%)	Sen (%)	ASD (mm)
**Tooth**	Ours (*w/o* S)	91.8	91.6	0.19	91.1	89.9	0.24
	Ours (*w/o* M)	92.7	91.9	0.23	91.4	90.3	0.29
	Ours	94.1	93.9	0.17	92.5	92.1	0.21
**Bone**	Ours (*w/o* H)	91.8	89.4	0.54	90.2	88.3	0.67
	Ours	94.5	93.8	0.33	93.8	93.5	0.40

Ours (*w/o* S): our AI system without skeleton information in the inputs of the 2nd-stage tooth segmentation network.
Ours (*w/o* M): our AI system without the multi-task learning scheme, and only predicting the tooth mask of the 2nd-stage tooth segmentation network.
Ours (*w/o* H): our AI system without the harr filter enhancement for bone segmentation.

Supplementary Table 3. Segmentation results of different methods trained on limited or large dataset, while tested on the external dataset with various abnormalities.

Methods	Training set (cases)	Dice (Tooth) (%)	Dice (bone) (%)
ToothNet*[1]	100	84.9	-
	100+	85.4	-
	3172	90.6	-
MWTNet*[2]	100	83.7	-
	100+	85.6	-
	3172	90.4	-
CGDNet*[3]	100	85.8	-
	100+	87.2	-
	3172	91.0	-
Ours	100	87.6	88.0
	100+	88.3	88.9
	3172	92.5	93.8

"*" means the method needs manual intervention (i.e., manually delineate foreground dental ROI). "+" means data argumentation techniques are used, including image flip, rotation, random deformation, and the learning based generative model.

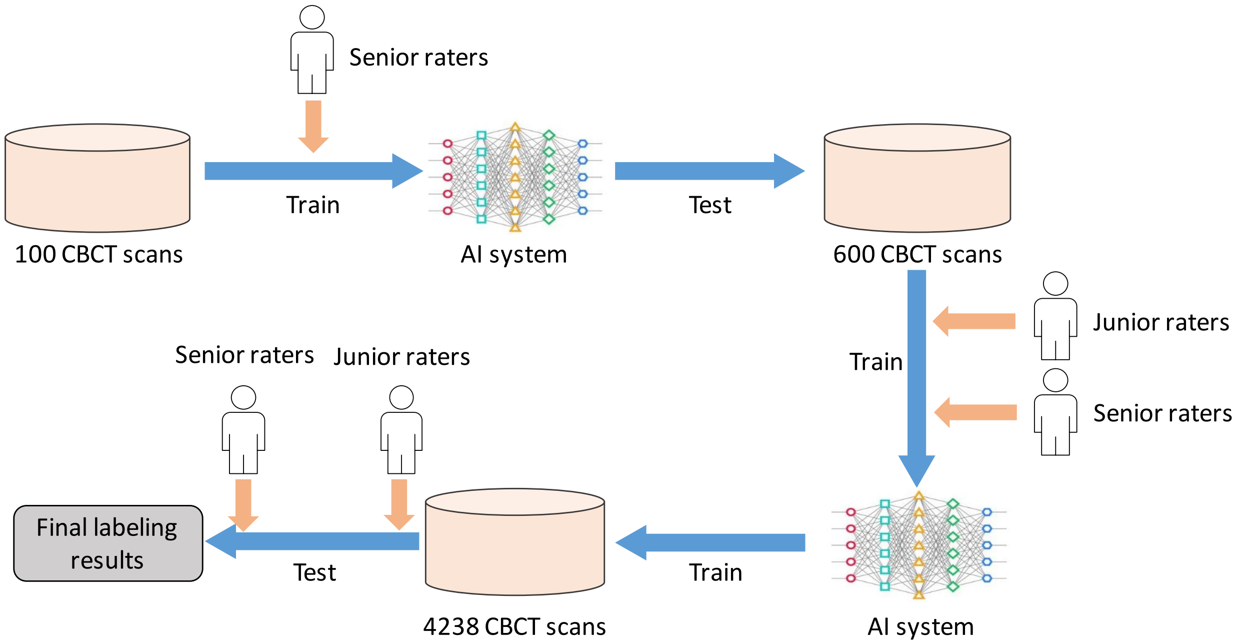

Supplementary Figure 1. The work flow of our hierarchical data annotation process.

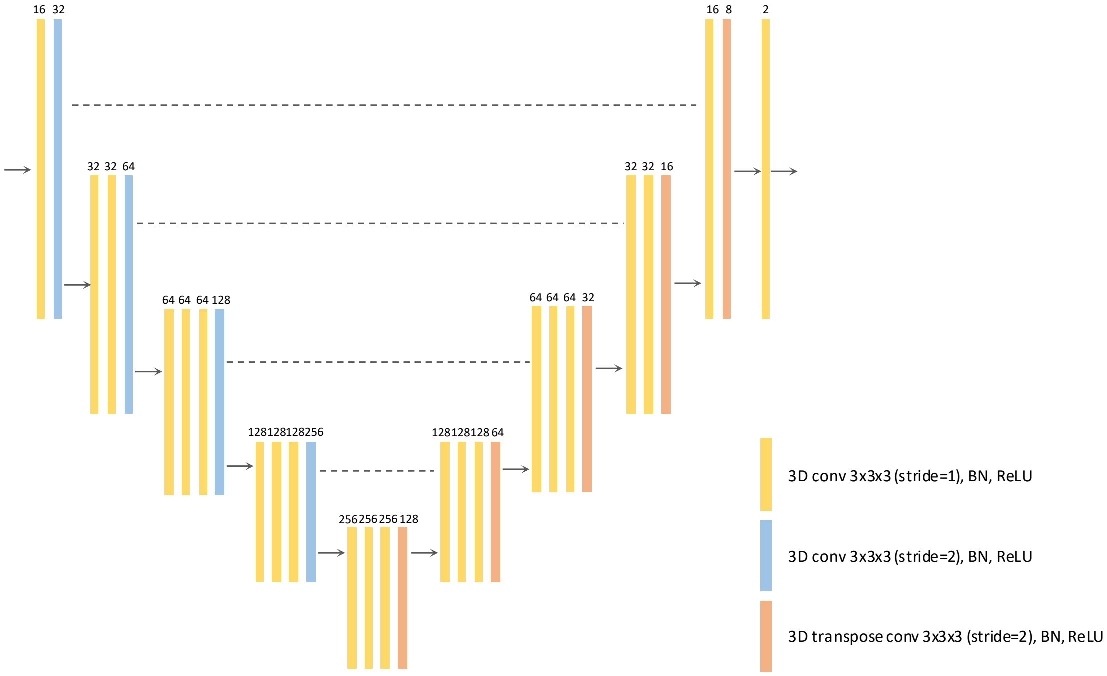

Supplementary Figure 2. The detailed network architecture of the ROI extraction network.

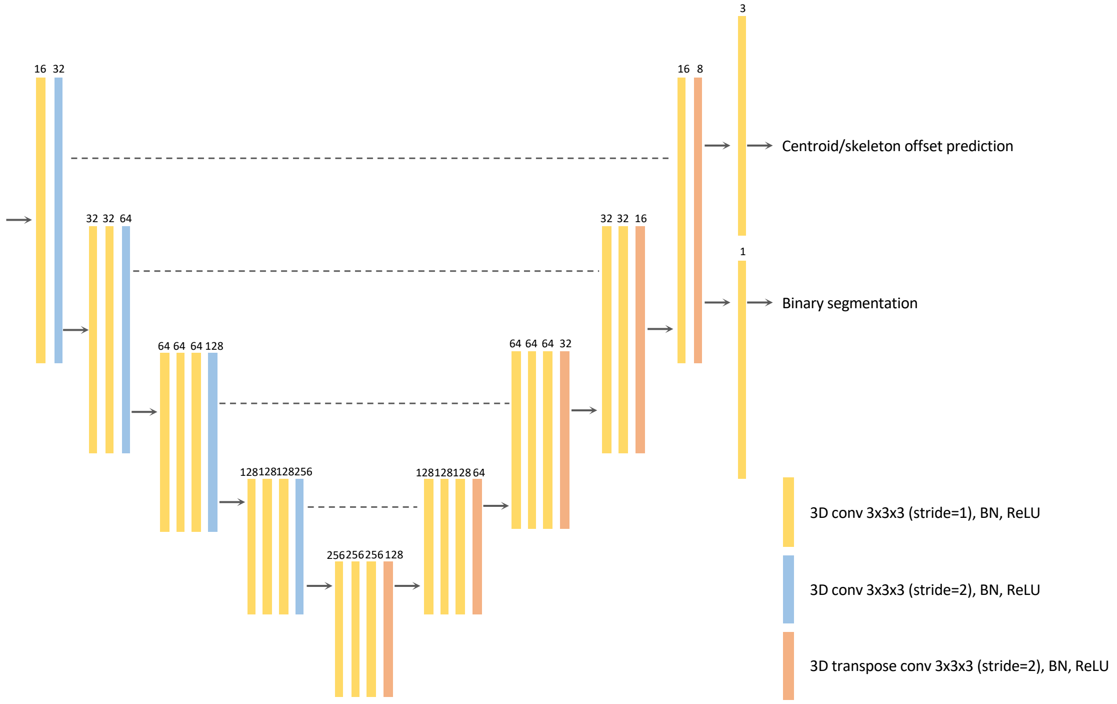

Supplementary Figure 3. The detailed network architecture of the tooth centroid or skeleton prediction network.

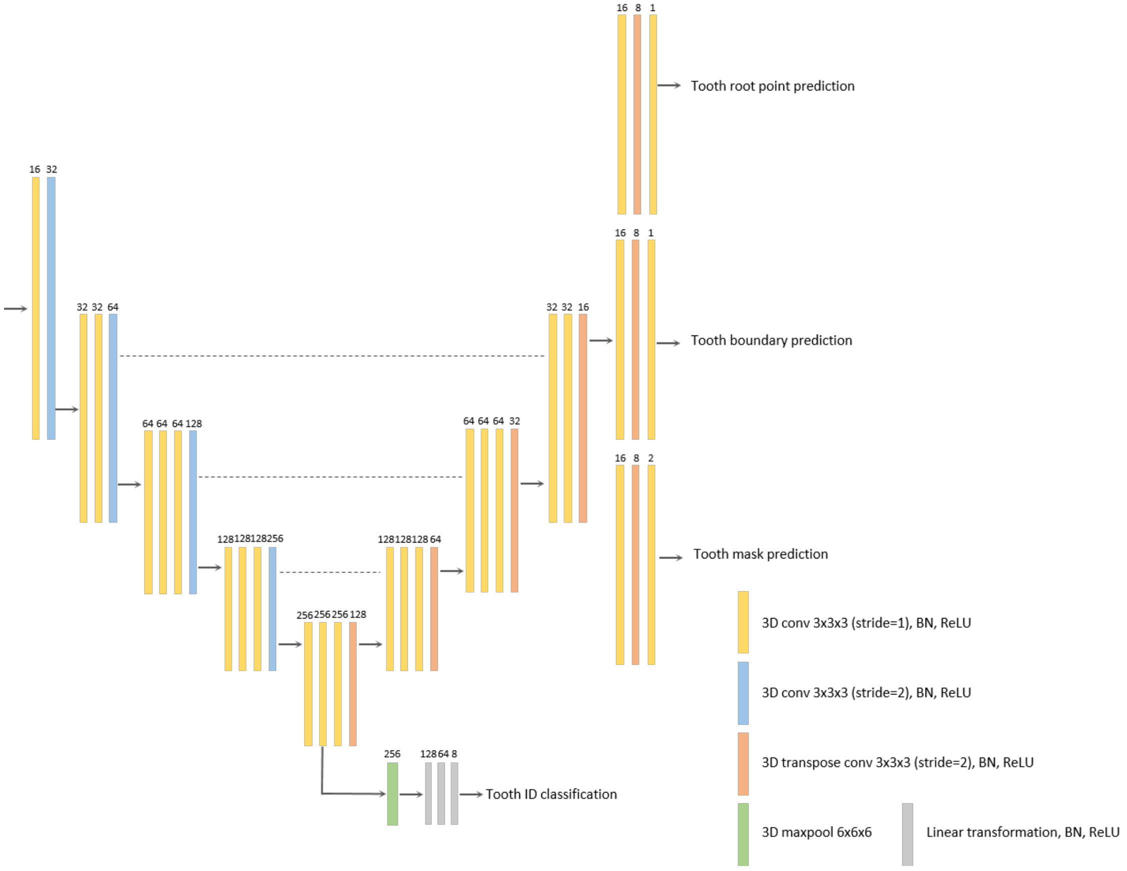

Supplementary Figure 4. The detailed network architecture of the multi-task tooth segmentation network.

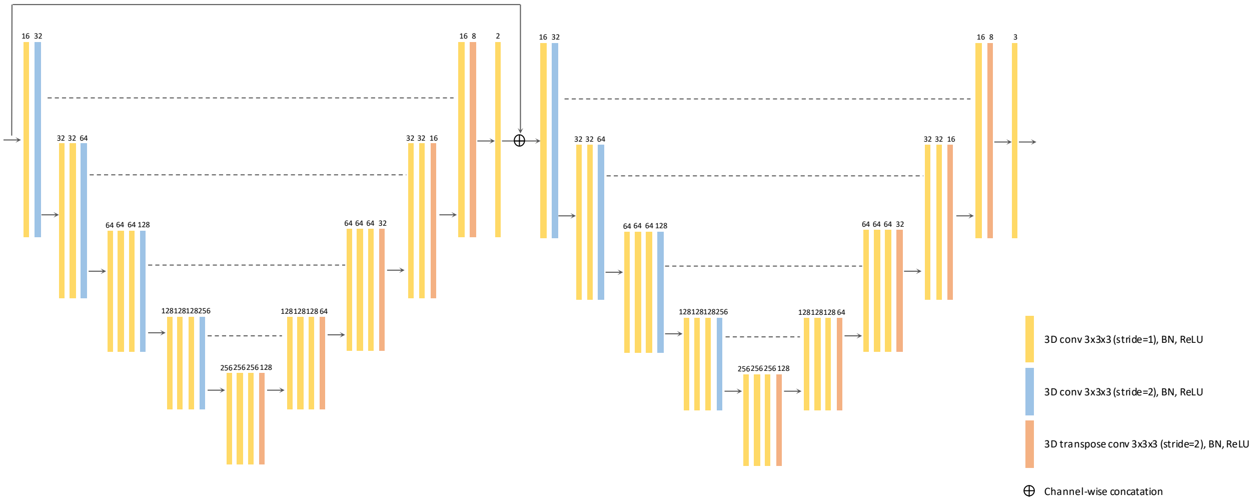

Supplementary Figure 5. The detailed network architecture of the cascaded bone segmentation network.

References

1. Cui, Z., Li, C. & Wang, W. Toothnet: Automatic tooth instance segmentation and identification from cone beam ct images. In *Proceedings of the IEEE/CVF Conference on Computer Vision and Pattern Recognition*, 6368–6377 (2019).

2. Chen, Y. *et al.* Automatic segmentation of individual tooth in dental cbct images from tooth surface map by a multi-task fcn. *IEEE Access* (2020).

3. Wu, X. *et al.* Center-sensitive and boundary-aware tooth instance segmentation and classification from cone-beam ct. In *2020 IEEE 17th International Symposium on Biomedical Imaging (ISBI)*, 939–942 (IEEE, 2020).
